# 10th Jubilee Multidisciplinary International Conference of Neuroscience and Biological Psychiatry “Stress and Behavior”

**DOI:** 10.1155/2007/70929

**Published:** 2007-09-03

**Authors:** Allan V. Kalueff

**Affiliations:** Center for Physiology and Biochemical Research (CPBR), 6-5 Cheshskaya Street, 01042 Kiev, Ukraine

## Abstract

St. Petersburg (Russia) hosted the 10th Jubilee Multidisciplinary Conference “Stress and Behavior” during May 16–20, 2007. The conference featured many foremost researchers speaking on recent developments on topics such as the role of neural plasticity, memory, learning, genetics, neuromediators, transporters, and steroids in stress research, spanning disciplines from fields ranging from neurogenetics to clinical psychiatry. The conference was attended by 700 delegates from over 40 nations.

The 10th Jubilee Multidisciplinary Conference “Stress and Behavior” was held in St. Petersburg, Russia, between 16–20 May, 2007. The conference was organized by the Centre for Physiology and Biochemical Research (CPBR), the Russian Society for Biopsychiatry (RSBP), Ukrainian Society for Biological Psychiatry (USBP), and the Institute of Experimental Medicine (IEM).

The conference's traditional topics included neurobiology and genetics of stress, role of neuromediators, transporters and steroids in the CNS, neurophysiology and experimental models of stress-evoked behaviors, the role of neural plasticity, memory and learning in stress, neuropsychology, psychoneuroimmunology, clinical psychiatry, neuroethology, as well as biomarkers of stress, gene x environment interactions, and translational research in biological psychiatry. Altogether there were 700 registered delegates representing 40 countries worldwide.

Opening the meeting, conference organizers emphasized the importance of biological markers of stress in behavioral research, the key role of cognitive factors, and important contribution of H. Selye, whose 100th anniversary since birth (1907) coincided with the conference. Reviewing a 10-year history of multidisciplinary “stress and behavior” conferences, their contribution to promoting translational research and international collaboration in neuroscience has been acknowledged. The establishment of a new professional scientific society—the International Stress and Behavior Society (ISBS)—was also announced.

The opening ceremony was followed by morning plenary lectures. Academy Professor R. Naatanen (Finland) overviewed the utility of mismatch negativity in biological psychiatry, Dr. A. V. Kalueff (USA) presented recent data on genetic animal models of serotonergic dysregulation, and Professor M. Issidorides (Greece) spoke on cell structural abnormalities in Parkinson's disease. After the lectures, there were parallel morning symposia on experimental models of stress and clinical psychology and psychiatry.

Day 1 afternoon, plenary lectures were given by Professors V. M. Klimenko (Russia) on biofeedback correction of adolescent deviant behaviors, and G. Fornaciari (Italy) on hydrocephaly at Medici's Court in Florence in the 16th century. Academy Professors M. Aghajanov and L. Mkrtchyan (Armenia) discussed ways of prevention of neurodegenerative disorders. Professors T. Sollertinskaya (Russia) and A. Weissman (Israel) focused on behavioral effects of peptides on stress-related behaviors across species, and on steroid-NO interactions. Plenary lectures were followed by continuing parallel symposia on experimental models of stress and clinical psychology and psychiatry. Social program of Day 1 included welcoming reception and panoramic city tour.

Day 2 morning, plenary lectures were given by Professors Z. Zukowska (USA) on the role of neuropeptide Y in the regulation of behaviors, A. Egorov (Russia) on phenomenology of addiction, and J.-T. Zhang (China)—Honored President of Chinese Pharmacological Society—who comprehensively evaluated antistress effects of ginsenoside compounds. Morning scientific symposia included symposium on biofeedback (organized by Professors E. Lyskov and B. von Scheele, Sweden) and symposium on clinical psychiatry.

Starting afternoon plenary lecture session, Professor M. Segal (Israel) focused on clinical comorbidity between posttraumatic stress and attention deficit-hyperactivity disorders. Professor P. D. Shabanov (Russia) discussed corticotrophin-releasing hormone and its role in the regulation of stress, and Professor Yu. F. Pastuhov (Russia) reviewed behavioral and physiological effects of heat shock proteins. A special symposium on psychoneuroendocrinology of stress, dedicated to Professor Sergei A. Chepurnov (1936–2007), and symposium on psychopharmacology finished the afternoon session. Social program of Day 2 included a boat trip along the rivers and canals of St. Petersburg.

Day 3 plenary lectures were delivered by Professors P. Seibert (USA), who spoke on CNS damage and its psychiatric consequences, A. Avital (Israel), on neonatal models of stress, and N. Enginar (Turkey), presenting recent data on psychopharmacology of rodent grooming and anxiety. Dr. O. Medina (Spain) discussed a spectrum of psychiatric disorders exaggerated in victims of the March 11th attack in Madrid. Scientific program of Day 3 also included symposia and round tables on translational research in stress neuroscience, animal models of stress, and behavioral and biological markers of stress. Social program of Day 3 included a conference dinner with traditional Russian cuisine, and inspiring folk songs and gypsy music.

Day 4 of the conference was held in the Institute of Experimental Medicine, hosted by I. Pavlov's Department of Physiology. There were plenary lectures on autoimmune CNS disorders (Dr. I. Abdurasulova, Russia) and domain interplay in behavioral phenotyping research (Dr. A. Kalueff, USA), followed by a poster session and a half-day Neuropsychoimmunology symposium.

Day 4 social program consisted of two parallel concerts: “Le Bayadere” ballet at the historic Mussorgsky Theatre, and music performance at St. Petersburg State Philarmony. Next day, there was a half-day guided tour to Peterhof Palace and gardens, and the 10th “Spa” symposium on biological psychiatry. This traditional evening satellite symposium gathered 20 scientists from 10 countries to discuss the role of cognitions in stress-related neuropsychiatric disorders.

Overall, the aim of the conference was to facilitate interdisciplinary dialogue, promote international collaboration, and encourage scientists from all over the world to attend the meeting. The audience consisted of psychiatrists (65%) and neurobiologists (35%). Following a tradition, 35% of the conference participants were young scientists and students. The organizing committee has awarded a number of travel fellowships to support their participation in the conference.

Concluding the conference, it was announced that the 11th Multidisciplinary Conference “Stress and Behavior”—the 1st ISBS conference—will be held in May 16–20, 2008 in St. Petersburg, Russia. In addition, the 1st ISBS Summer School on behavioral genetics and neuroscience will be organized between May 9–15, 2008, in conjunction with the 2008 meeting.

